# Neutrophils in Health and Disease: From Receptor Sensing to Inflammasome Activation

**DOI:** 10.3390/ijms24076340

**Published:** 2023-03-28

**Authors:** Agnieszka Iwaniuk, Ewa Jablonska

**Affiliations:** Department of Immunology, Medical University of Bialystok, 15-269 Białystok, Poland

**Keywords:** neutrophil, inflammasome, pyroptosis, cardiovascular disease, COVID-19, CAPS

## Abstract

Neutrophils—polymorphonuclear cells (PMNs) are the cells of the initial immune response and make up the majority of leukocytes in the peripheral blood. After activation, these cells modify their functional status to meet the needs at the site of action or according to the agent causing injury. They receive signals from their surroundings and “plan” the course of the response in both temporal and spatial contexts. PMNs dispose of intracellular signaling pathways that allow them to perform a wide range of functions associated with the development of inflammatory processes. In addition to these cells, some protein complexes, known as inflammasomes, also have a special role in the development and maintenance of inflammation. These complexes participate in the proteolytic activation of key pro-inflammatory cytokines, such as IL-1β and IL-18. In recent years, there has been significant progress in the understanding of the structure and molecular mechanisms behind the activation of inflammasomes and their participation in the pathogenesis of numerous diseases. The available reports focus primarily on macrophages and dendritic cells. According to the literature, the activation of inflammasomes in neutrophils and the associated death type—pyroptosis—is regulated in a different manner than in other cells. The present work is a review of the latest reports concerning the course of inflammasome activation and inflammatory cytokine secretion in response to pathogens in neutrophils, as well as the role of these mechanisms in the pathogenesis of selected diseases.

## 1. Mechanisms of Recognition and Killing of Pathogens by Neutrophils

Neutrophils—polymorphonuclear granulocytes (PMNs) are the first line of defense against pathogenic microorganisms and cancer cells. PMNs also play the role of regulatory cells in the mechanisms of the acquired immune response [1]. Detection of invading microbes is via pathogen-associated molecular patterns (PAMPs) through Toll-like receptors (TLRs). Neutrophils express the majority of TLR family members, lacking only the intracellular receptors such as TLR3 and TLR7. Since the main role of neutrophils is to defend against bacteria, it is the TLRs located on the cell surface that are the most well-studied in neutrophil TLR biology [2]. However, the intracellular TLRs may also play important roles in response to the pathogen. TLRs also have the ability to detect endogenous molecules called damage-associated molecular patterns (DAMPs) that are produced by cells in response to injury or infection [3]. TLR activation leads to important cellular processes, including phagocytosis, reactive oxygen species (ROS) and nitric oxide (NO) generation, cytokine production, and increased survival, all of which can contribute to the pathogenesis of chronic inflammation [4,5,6]. 

Another weapon that neutrophils have in their anti-pathogenic arsenal is the release of neutrophil extracellular traps (NETs). The traps formed from DNA fibers, histone proteins, and serine proteases (mainly neutrophil elastase and myeloperoxidase) derived from the granules are released outside the cell. The release of these structures is intended to immobilize pathogens, including Gram-positive and Gram-negative bacteria, fungi, protozoa, and viruses. In addition, NETs constitute a physical barrier that facilitates the degradation of bacterial and viral virulence factors and protects the host organism against the spread of pathogenic microorganisms [7,8]. The consequence of recognizing the pathogen and performing many effector functions by neutrophils is the development of the inflammatory process due to the secretion of pro-inflammatory cytokines by these cells. A significant cytokine secreted by PMNs, contributing to the development of the inflammatory process by recruiting and activating inflammatory cells, is IL-1β. It has been shown that the secretion of IL-1β requires the activation of inflammasomes—multimeric complexes whose activation is triggered by pathogenic or non-pathogenic signals [9,10].

## 2. Inflammasomes—Structure and Activation

Pattern recognition receptors (PRRs) belong to numerous families, which can be classified based on their location in the cell, structural construction, or function. Some of these, including NOD-like receptors (e.g., NLRP3), act as intracellular “sensors”, receiving information on the “danger” and activating the appropriate immune response adapted to the antigen type [11,12].

Thus far, the best-studied signaling mechanism is NLRP3 inflammasome activation described for monocytes/macrophages. This multi-protein complex includes the intracellular NLRP3 receptor NALP (nucleotide-binding oligomerization domain, leucine-rich repeat, and pyrin domain-containing)/cryopyrin, ASC (apoptosis-associated speck-like protein containing a CARD (Caspase activation and Recruitment Domain) adaptor protein, and procaspase-1 [13]. 

**Figure 1:**
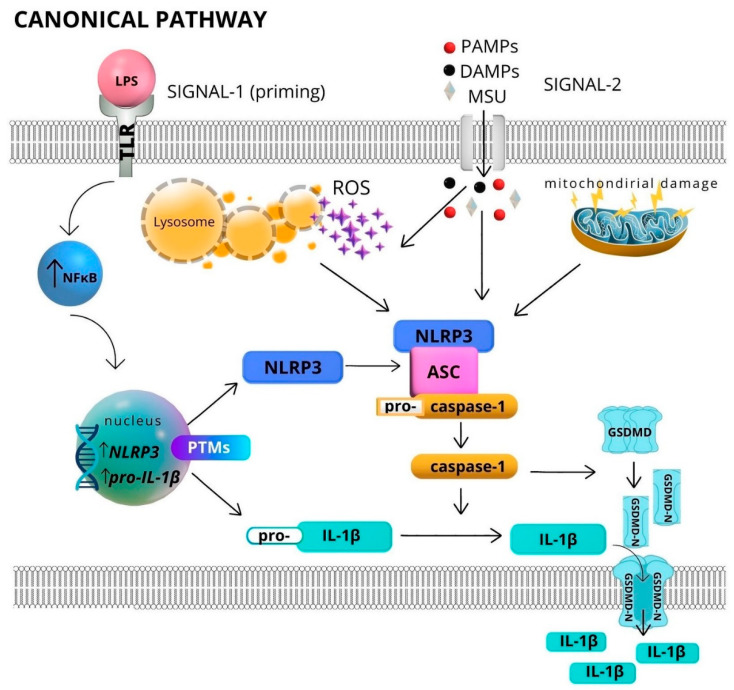
Formation of NLRP3 inflammasome and its activation through the canonical pathway. Abbreviations: PAMPs—pathogen-associated molecular patterns; DAMPs—damage-associated molecular patterns, MSU—crystals of monosodium urate; TLR—toll-like receptors; NF-kB—nuclear factor kappa-light-chain-enhancer of activated B cells; PTMs—post-translational modifications; ROS—reactive oxygen species; NLRP3—NLR family pyrin domain containing 3; ASC—apoptosis-associated speck-like protein containing a CARD; GSDM- gasdermin D; GSDMD-N—N-end fragment of gasdermin D. Signal 1 causes priming through the activation of NF-κB [16]. The action of this nuclear factor in the cell nucleus results in increased transcription of pro-IL-1β and NLRP3 genes and a series of posttranslational modifications, including phosphorylation, acetylation, and ubiquitination, enabling the NLRP3 protein to assume the appropriate conformation [25,26,27]. Signal 2, which is triggered by numerous extracellular stimuli, including danger-associated molecular patterns, pathogen-associated molecular patterns, or uric acid crystals, leads to the binding of NLRP3, ASC, and pro-caspase-1. Other intracellular signals may also contribute to the activation of inflammasomes, through the action of signal 2, lysosome burst, release of reactive oxygen species, or mitochondrial damage. In the next stage, pro-caspase-1 bound in the inflammasome complex undergoes autoproteolytic activation. Caspase-1 induces the proteolytic activation of pro-IL-1β to its active form and cleaves the N-end fragment of gasdermin D, which accumulates in the cell membrane, creating pores and enabling the release of IL-1β to the extracellular environment [19,28].

The canonical activation of the NLRP3 inflammasome requires two signals. The first signal (signal 1) is transcriptional priming resulting from ligand action for TLR (e.g., bacterial lipopolysaccharide [LPS] and TLR4) to increase the expression of NLRP3 and pro-IL-1β via NF-κB [14,15,16]. The second (signal 2) is a broad spectrum of infectious and noninfectious signals related to cellular stress (PAMPs: bacterial toxins, pathogen RNA; DAMPs: ATP, K^+^ ionophores, crystals) [17,18,19,20,21]. The N-end of NLRP3 containing the PYD effector domain (pyrin domain) binds to the homologous domain of the ASC protein. This results in the spontaneous oligomerization of ASC in the cytosol and subsequently the formation of long, filamentous structures, which organize into macromolecular complexes known as ASC spots. On the C-end of the ASC protein, CARD binds to the p20 subunit of procaspase-1 via a homologous interaction. As a result, procaspase-1 is cleaved into its active form, causing proteolytic cutting of pro-IL-1β to active IL-1β. Moreover, caspase-1 has the ability to activate Gasdermin D (GSDMD). The N-end fragment of GSDMD formed after proteolytic activation penetrates the plasma membrane and ultimately leads to pore formation, cellular lysis, and IL-1β release into the extracellular space [22,23]. When a cell is subjected to these processes, it develops an inflammatory phenotype, as observed in the lytic, inflammatory caspase-1-dependent form of death known as pyroptosis [24]. The canonical activation of the NLRP3 inflammasome is shown in Figure 1.

**Figure 2:**
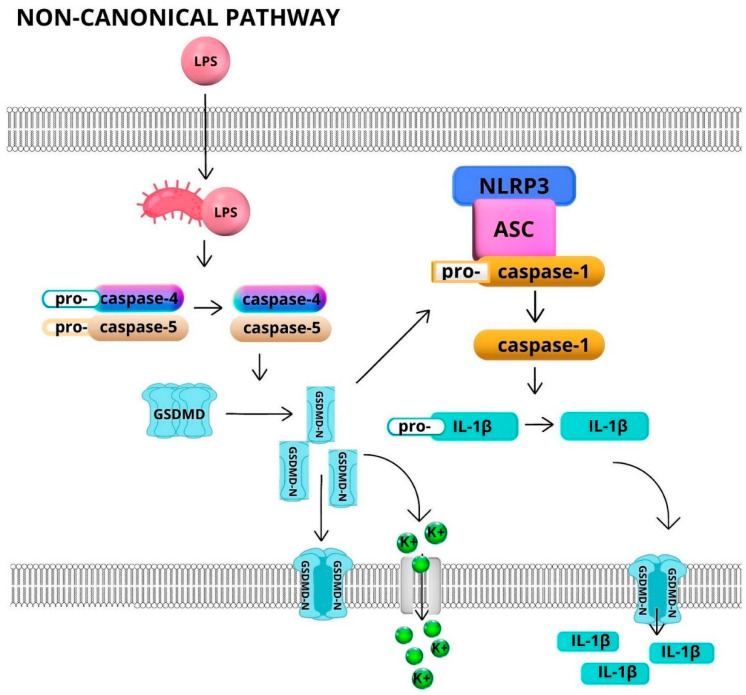
Non-canonical activation of NLRP3 inflammasomes. Abbreviations: LPS—lipopolysaccharide; NLRP3—NLR family pyrin domain containing 3; ASC—apoptosis-associated speck-like protein containing a CARD; GSDMD—gasdermin D; GSDMD-N—N-end fragment of gasdermin D. The non-canonical inflammasome pathway is activated by intracellular (e.g., in *Escherichia coli* infections) or extracellular (circulating) LPS. LPS has the ability to induce direct proteolytic activation of procaspase-4 and procaspase-5. Activation of inflammasome occurs due to the efflux of K^+^ ions from the cell or the cleavage of gasdermin D through the effect of active caspase-4 and caspase-5 [30,31].

NLRP3 inflammasome is also activated by other mechanisms. Its non-canonical activation occurs in response to bacterial or circulating cytosolic LPS. As a consequence, caspase-11 (in mice) or caspase-4 and caspase-5 (in humans) causes GSDMD cleavage. The pores formed by the N-end fragment of GSDMD allow the efflux of K^+^ ions, resulting in the canonical activation of NLRP3 following caspase-1 cleavage [29]. Non-canonical activation of the NLRP3 inflammasome is shown in Figure 2.

### Activation of Inflammasomes in Neutrophils

AIM-1 receptor, which is classified among ALRs (absent in melanoma-2 (AIM2)-like receptors), and receptors from the family of NLRs (NLRP1, NLRP3, NLRC4, NLRP12) have been recognized so far as receptors of neutrophils participating in the formation of inflammasomes, whose expression has been confirmed at the mRNA and protein level [32]. Proteins forming inflammasomes can be found in neutrophils, within several cellular compartments. Such proteins have also been observed in the cytoplasm, as well as in the secretory vesicles and grade III granules. The presence of *caspase-1, AIM-2, ASC, NLRP1, NLRP3,* and *NLRC4* has been confirmed at the mRNA level. Moreover, the expression of *NLRP3* and *NLRC4* was observed both in human and mouse neutrophils at a similar or higher level compared to other types of cells, such as bone-marrow-derived macrophages or bone-marrow-derived dendritic cells [9,33]. It was shown that the expression of the *NLRP3* gene was increased in mouse neutrophils under inflammation induced in vivo with TNF and muramyl dipeptide, and a lower expression was observed in mouse resting neutrophils [34]. Priming of human peripheral blood neutrophils with LPS led to elevated expression of *IL-1A* and *NLRC4* genes. A Western-blot-based study on the expression of inflammasome-forming proteins following exposure to LPS confirmed that ASC, AIM-2, and caspase-1 were found in human neutrophils, demonstrating the presence of NLRP3 protein in these cells [9]. 

In another study, immunofluorescent staining confirmed the role of inflammasome-forming proteins in the activation of neutrophils. The study also reported:

1. The presence of numerous spots of enzymatically active caspase-1 (confirmed with a FLICA test) in the PMNs of the infected cornea; and
2. ASC staining in bone-marrow-derived neutrophils stimulated with thermally inactivated *Streptococcus pneumoniae* and pneumolysin (confirmed by positive FLICA test and fluorescence), indicating the presence of NLRP3.

Moreover, the presence of high-molecular-mass ASC corresponding to the oligomers of NLRP3 has been reported [35]. However, the expression of caspase-1 and ASC adapter protein was found to a lesser degree than in macrophages [36].

In conclusion, the presented data seem to confirm that the activation of inflammasomes and the mechanisms responsible for the secretion of IL-1β have a different course in neutrophils than in macrophages. The main differences are: “support” of the canonical pathway by serine proteases, secretion of IL-1β without lytic death—pyroptosis, a different (still not fully explored in neutrophils) mechanism of action of Gasdermin D, which simultaneously supports the secretion of IL-1β and provokes the release of NETs. We will discuss these issues in detail in the following sections.

## 3. Expression of IL-1β by Neutrophils with the Contribution of Inflammasomes

Because neutrophils are abundant in both circulating and marginal pools due to recruitment by inflammatory mediators, physical effort, or the activity of adrenaline, these cells promote inflammation by secreting chemokines and cytokines [37]. IL-1β is the primary inflammatory regulator controlling the innate immune processes and performing various biological functions. Its action includes the induction of acute inflammation phase components and pyrogenic activity in cells and the central nervous system. Moreover, this cytokine plays a significant role in regulating adaptive response through the recruitment and activation of T and B lymphocytes [38,39,40]. 

It should be underlined that efficient activation of inflammasomes in cells results in the proteolytic activation of IL-1β and its expression outside the cell. Currently, several research groups are investigating the expression of IL-1β by neutrophils with an aim of understanding the precise mechanism. However, previous reports clearly indicate that the process of IL-1β expression by neutrophils differs from that by monocytes or macrophages [41]. 

### 3.1. Inflammasome Activation with Canonical Pathway and Serine Proteases

Studies on macrophages have shown that caspase-1 is an important element in the process of IL-1β maturation [42]. However, the results from a study on neutrophils by Greten et al. cast doubt on the need for the participation of caspase-1 and, thus, other inflammasome-forming proteins in the maturation of proteolytic IL-1β [43]. The study revealed the contribution of serine proteases, mainly proteinase-3 and, to a lesser extent, neutrophil elastase, in the maturation of IL-1β in PMNs. Another study conducted by Mankan et al. on a mouse model allayed this doubt and proved that neutrophils lacking the *caspase-1* gene did not secrete IL-1β [44]. Moreover, NLRP3 protein, ASC, and caspase-1 were shown to be essential for the production of IL-1β and bacterial lysis by neutrophils in a mouse model of corneal infection with *S. pneumoniae* [35]. The above research confirmed that the canonical inflammasome activation pathway is of importance in neutrophils. A study conducted several years later, however, confirmed that serine proteases play a significant role in the expression of IL-1β in neutrophils. This suggests that although caspase-1 is necessary for the expression of this cytokine, it is rapidly inactivated, and only neutrophil serine proteases are key components in the processing of pro-IL-1β into its mature form. According to the authors of the cited studies, the regulatory role of serine proteases in IL-β expression probably indicates their therapeutic applicability. Inhibition of serine proteases can be a more efficient approach compared to therapies targeting IL-1β alone in pathological conditions caused by this cytokine derived from neutrophils [45]. 

### 3.2. Neutrophils vs. Pyroptosis—Nonlytic Expression of IL-1β

Kramakar et al. made a significant discovery regarding the differences between macrophages and neutrophils. Their studies showed that PMNs avoid pyroptosis by expressing mature IL-1β [26,37]. The authors reported that neutrophils in which activation of the NLRC4 inflammasome occurred by transfection of bacterial flagellin or infection with Salmonella bacteria expressed IL-1β. The maturation of pro-IL-1β to its mature form was conditioned by caspase-1, while secretion of this cytokine in the early phase was dependent or partially dependent on GSDMD. However, the mechanism of secretion changed over time. Three hours after the activation of the inflammasome, the release of IL-1β from the cell occurred independently of GSDMD [22,38]. Differences in the inflammasome signaling pathway between neutrophils and macrophages seem to be important in the context of antimicrobial functions performed by neutrophils. In contrast to macrophages, non-lytic expression of IL-1β enables neutrophils to maintain the concentration of this cytokine at the site of infection and perform protective functions [39].

### 3.3. Non-canonical Activation of Inflammasome in Neutrophils—IL-1β and NETs

Kaiwen et al. proposed the non-canonical model of IL-1β release in neutrophils, which provided considerable data regarding the pyroptosis-avoiding phenomenon of neutrophils. These authors found that under the influence of cytosolic LPS and Gram-negative bacteria, GSDMD was more efficiently cleaved in PMNs by caspase-11 than caspase-1, which is markedly less expressed in neutrophils than in macrophages. This resulted in the formation and ejection of neutrophil extracellular traps (NETs) and neutrophil NETotic cell death [46]. Almost at the same time, another group of researchers confirmed the involvement of GSDMD in the formation of NETs [47]. Going one step further, the authors proposed that the activity of caspase-1 in neutrophils is low enough to allow sublytic cutting of GSDMD, not only to facilitate the expression of IL-1β but also to prevent pyroptosis. Moreover, they hypothesized that NETotic cell death may be a mode of cell death specific for signaling neutrophils via non-canonical inflammasome activation [46].

### 3.4. IL-1β Expression with Contribution of Autophagosomes

The latest reports on the expression of IL-1β combine many of the above-discussed results and emphasize cellular identity and the associated differences in intracellular signaling and IL-1β expression between macrophages and neutrophils. Since IL-1β lacks the signal sequence required for conventional expression by the endoplasmic reticulum, this cytokine requires nonconventional mechanisms participating in its release outside of the cell [48,49]. A study by Vijayaraj et al. proved that the intracellular pool of pro-IL-1β cannot be effectively and directly cleaved by caspase-1 until it undergoes a “checkpoint” in the form of post-translational modification—ubiquitination. Deubiquitination of lysine K133 caused by the presence of LPS promotes IL-1β turnover, increases the level of pro-IL-1β, and enables proteolytic cleavage of caspase-1 to the mature form of IL-1β [50]. Gasdermin D participates, in the classical mechanism described in macrophages, in the secretion of IL-1β outside the cell. The inactive GSDMD precursor consists of an N-terminal domain (p31 GSDM-N), a linker region, and a C-terminal autoinhibitory domain (p24 GSDM-C). Gasdermin D activation is initiated by proteolytic cleavage in the linker region, and p31 GSDM-N fragments integrate into the cell membrane by forming pores, inducing pyroptosis, and releasing IL-1β out of the cell [51,52]. It was found that in neutrophils, contrary to macrophages, the p31 N-GSDMD fragment, which is capable of forming pores in the cell membrane, did not move efficiently in the vicinity of the cell membrane following LPS/ATP activation. Rather, the fragment bound to the membranes of neutrophil azurophilic granules, causing the release of neutrophil elastase into the cytosol, where this enzyme took part in the cleavage of GSDMD and formation of the p24 C-GSDMD fragment. The above study also demonstrated the connection of the p31 N-GSDMD fragment with LC3^+^ autophagosome membranes [53]. The relationship between autophagy and IL-1β expression by neutrophils has also been reported earlier. Pharmacological inhibition of autophagy, as well as ATG5 knockdown, reduced the amount of active caspase-1, whereas stimulation of autophagy via starvation resulted in an increased amount of this enzyme [54]. This likely stems from both increased enzyme activation, as well as limited removal of inflammasomes by autophagy [45,55]. Keitelman et al. hypothesized that inflammasome removal by autophagy has different effects in macrophages and neutrophils; in macrophages, inflammasome removal may inhibit the expression of IL-1β, while in neutrophils, it may limit the expression of serine proteases from azurophilic granules, avoiding IL-1β degradation. However, this presumption requires confirmation through further research [45].

## 4. Inflammasomes in Neutrophils and Their Contribution to Disease Pathogenesis

From the standpoint of antimicrobial response, inflammasomes represent the contact point of innate and acquired immune mechanisms. Their activation following pathogen “detection” is crucial for the modulation of adaptive response against the pathogen. As shown by various research groups, the functions of neutrophils participating in antimicrobial response vary depending on the immunological context. As the first line of defense, these cells infiltrate the pathogen penetration site where high levels of DAMPs (damage-associated molecular patterns) are found signaling the danger from damaged cells [56]. Despite their short lifespan, neutrophilic granulocytes exhibit prolonged antimicrobial action, which is harmful to cells and tissues of the host [10]. 

Inflammasome activation is now considered responsible for the development of numerous inflammation-based diseases. Given that neutrophils avoid pyroptosis during IL-1β expression, unlike macrophages, these cells are assumed to play a key role in the prolonged inflammatory process. This hypothesis was confirmed by the study by Son et al., which showed that the inflammatory microenvironment rich in DAMPs reversed the NLRP3 activation potential in macrophages but not in neutrophils. The authors indicated that this was linked to the polarization of mitochondria by DAMPs in macrophages, and it did not occur in neutrophils due to the lack of SARM-1 (a negative regulator of TLR signaling) expression [57,58]. Moreover, it was shown that neutrophils did not cause polarization of macrophages to M2, neutralizing inflammation through spherocytosis [57,59]. The prolonged inflammasome activation potential of neutrophils may contribute to the pathogenesis of chronic inflammatory conditions, which are mediated by these cells. 

### 4.1. Cardiovascular Diseases

Inflammatory lesions caused by neutrophils have long been a topic of interest and the reference point for the treatment of cardiovascular diseases such as acute coronary syndrome, deep venous thrombosis, and heart failure [60,61]. The role of neutrophils in the pathogenesis of cardiovascular diseases is highlighted by research assessing neutrophil count as a risk factor for complications in the course of these diseases or for sudden death. Patients with different types of cardiovascular diseases who had neutrophil counts in the upper normal limit were more likely to have an unexpected coronary death, nonfatal myocardial infarction, heart failure, peripheral artery diseases, and abdominal aortic aneurysm compared to people with neutrophils in the lower normal limit [62]. 

**Figure 3:**
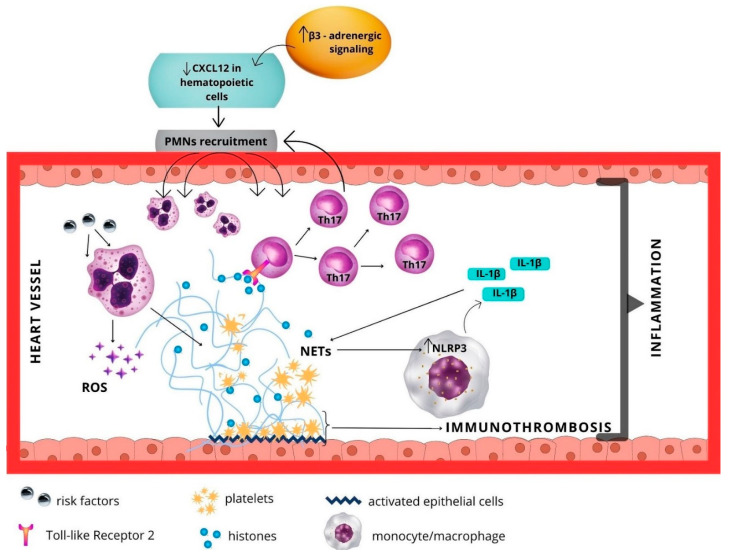
The mechanism of immunothrombosis and inflammation mediated by neutrophils in the blood vessel of the heart.

The contribution of PMNs in the pathogenesis of atherosclerotic disease or recovery following myocardial infarction is related to their sterile inflammatory response. Neutrophils activated by various risk factors of vascular diseases, including hyperglycemia, participate in degranulation, phagocytosis, production of reactive oxygen species (ROS), and NET release in the myocardium and peripheral and coronary vessels [63,64]. These cells also play an important role in NET-dependent immunothrombosis by interacting with vascular endothelial cells and platelets [65]. The expression of NETs and persistent inflammation by neutrophils in cardiovascular diseases is driven by other cells participating in the process, resembling a vicious cycle. Activated neutrophils produce significant amounts of ROS, followed by the release of NETs which activate the macrophage NLRP3 inflammasome. This protein complex expresses large quantities of IL-1β, which intensifies the expression of NETs, and in turn, NETs activate the NRLP3 inflammasome in macrophages [66]. Histones included in the NETs interact with TLR2 (Toll-like Receptor-2) on T cells, contributing to the phosphorylation of STAT3 and differentiation of T lymphocytes into Th17, which strongly recruits neutrophils. This ultimately results in prolonged and intensified neutrophil-dependent inflammation [64,67]. The mechanism of immunothrombosis and inflammation mediated by neutrophils in the blood vessel of the heart is presented in Figure 3.

During myocardial infarction, neutrophils are mobilized from the marrow due to increased signaling on β3-adrenergic receptors in the sympathetic system. This leads to a reduction in the expression of CXCL12 on hematopoietic cells and the release of PMNs to peripheral blood [68]. In a mouse model, a relationship between the presence of leukocyte-derived gasdermin and the increased recruitment of PMNs from the bone marrow at the early stage of myocardial infarction was found. Genetic (*GSDMD* −/− mice) and pharmacological (necro sulfonamide) inhibition of GSDMD reduced the number of PMNs and contributed to the clinical improvement by attenuating the infarct status, reducing infarct size, improving cardiac function, and increasing survival [69]. 

Neutrophils intensify the acute inflammatory response by responding first to molecular patterns released from damaged cardiomyocytes [70]. Their unfavorable activities include persistent inflammation and delayed repair of hypoxic myocardial tissues. It was found that among neutrophils infiltrating damaged myocardial tissues, approximately on the 3rd day after infarction, a subset of neutrophils with SighlecF^hi^ phenotype accumulated in the microenvironment of damaged tissues, while such cells were not seen in the peripheral blood. The profile of these cells predisposes them to increased phagocytic activity and ROS production [71]. This clearly indicates that the neutrophil aging process, which has a circadian rhythm under the physiological state, follows a different course than in myocardial infarction [72,73]. Persistent neutrophilic infiltration prolongs inflammation and the tissue repair process and may result in poor reconstruction of the left heart ventricle [74]. 

Approximately 5–7 days after infarction, inflammatory neutrophils (N1) polarized into a population of noninflammatory cells (N2), supporting the healing and regeneration of damaged myocardial tissues. Moreover, limited recruitment of PMNs and increased release of IL-4 by neutrophils in situ induced the proliferation of repairing M2 macrophages [75].

The diversity and complexity of processes involving neutrophils demonstrate the vast therapeutic potential of these cells in the treatment and prevention of cardiovascular diseases in high-risk individuals. Neutrophil-dependent inflammation in cardiovascular diseases has been investigated for a long time in studies focusing on the development of new therapies for these diseases. Clinical studies indicate that canakinumab—a human monoclonal anti-IL-1β antibody—showed efficiency in the secondary prevention of myocardial infarction [76]. Similarly, colchicine—an NLRP3 inflammasome inhibitor—showed efficiency in the secondary prevention of coronary ischemia [77,78].

### 4.2. Severe Course of COVID-19

The clinical course of SARS-CoV-2 infection is highly variable, with symptoms ranging from flu-like to severe (sometimes multiorgan) hypoxemia. Autopsy studies in patients with a severe course of COVID-19 showed elevated levels of pro-inflammatory cytokines (IL-1β and IL-6) in the serum and pulmonary tissue, as well as plasma [79,80]. Undoubtedly, the main clinical problem is uncontrolled inflammation, which occurs alongside a cytokine storm. Earlier research showed that inflammasomes and pyroptosis of monocyte play a role in inflammation during SARS-CoV-2 infection [81]. Since the beginning of the pandemic, scientists have been noticing the resurgence of neutrophilia, particularly in patients with a severe course of COVID-19 [82]. Studies on patients treated at the Wuhan Union Hospital in China revealed a markedly lower level of lymphocytes and an increased neutrophils/lymphocytes ratio in critically ill patients as compared with patients with the severe or mild disease [83]. Neutrophils made up between 79 and 90% of peripheral blood cells, outnumbering monocytes by about 10 times [84]. Granulocytes were also found in the tracheal aspirates of patients with a severe COVID-19 course, accounting for 80% of all myeloid cells CD45^+^, of which 60% were CD66b^+^ cells—neutrophils [85,86]. In addition to other serum markers, the count of PMNs in peripheral blood and the neutrophils/lymphocytes ratio was proposed as prognostic indicators of disease severity and even mortality [87,88]. Neutrophils from patients with COVID-19 exhibited ASC spot fluorescence, with the highest intensity in colocalization with NLRC4 protein (48% patients), followed by NLRP3 (28.9%), AIM-2 (15%), and NLRP1 (13.9%) [84]. A study on macrophages by Jaqueira et al. showed a significant correlation between the expression of quantitative trait loci (eQTL) and the severe course of *COVID-19* with the *NLRC4* gene, as well as between the eQTL of the *AIM-2* gene and the hospitalization frequency of COVID-19 patients with [89]. Considering the broad distribution of neutrophils (both in the blood and in the pulmonary tissue) in comparison with other leukocytes and the activation of inflammasomes in response to SARS-CoV-2 in patients with COVID-19, it can be presumed that both neutrophils and monocytes contribute to cytokine storm through inflammasome activation. 

A serious clinical problem related to the course of COVID-19 itself, as well as its complications, is frequent hemostatic disorders, which cause venous thromboembolic disorder in patients with COVID-19, or disseminated intravascular coagulopathy in those with an infection concomitant with COVID-19 [90]. Thromboembolic complications of SARS-CoV-2 infection are burdened by a high mortality risk. Research indicates that people who did not survive had a rapid increase in the levels of D-dimer [91]. Moreover, autopsy studies showed the presence of microthrombi in small pulmonary arteries and disseminated microvascular thrombi in numerous organs [92,93]. The contribution of NETs was described in the pathogenesis of various types of thrombosis, as extracellular traps favor the formation of thrombi by working as scaffolding, activating platelets, and inducing intravascular coagulopathy [94]. The development and activation of inflammasome were shown to play a significant role in NETs formation during sterile inflammation [95]. In patients with COVID-19, the percentage of oligomerized ASC protein spots in neutrophils indicated the presence of citrullinated histones (H3cit+) [84]. NETs were observed in plasma samples, tracheal aspirates, and pulmonary tissues from autopsies of COVID-19 patients. The potential for the formation and release of NETs was also increased in the neutrophils of COVID-19 patients as compared with healthy people. According to the research, live but not inactivated SARS-CoV-2 virus-induced neutrophils produce NETs. It was found that virus penetration inside neutrophils and its replication occurred through the fusion of virus spike protein (S) and the angiotensin-converting enzyme (ACE2) and the cleavage of S protein by TMPRSS2 serine protease, the expression of which was also confirmed in neutrophils [96,97]. Furthermore, evidence suggests that NETs form the scaffolding for thrombus bound to von Willebrand factor (VWF), which is cleaved by the ADAMRS13 enzyme [98]. In patients with a severe course of COVID-19, the concentrations of VWF exceeded the reference value by 500%. Moreover, a very high VWF/ADAMTS13 ratio was observed, which was likely due to intensive endothelial activity. Intensified VWF release may favor the retention of NETs in vessels [84]. In patients with a severe course of COVID-19, apoptotic processes in the cells of pulmonary epithelium and endothelium impeded the functional status of the lungs, resulting in a compromised state [99]. At the same time, it was demonstrated that neutrophils activated by SARS-CoV-2 and purified NETs from healthy neutrophils induced apoptosis in cells of the A549 line (cells isolated from lung tissue). These results indicate that NETs may be a potential factor damaging pulmonary epithelium cells in patients with severe SARS-CoV-2 infection. Moreover, NETs may activate PRR, including TLR-4 and TLR-7, which mediate the release of inflammatory mediators, intensifying the effect of NETs in patients with COVID-19 [97].

### 4.3. Cryopyrin-Associated Periodic Syndromes

The term “cryopyrin-associated periodic syndromes” (CAPS) collectively refers to a group of autoinflammatory diseases based on *NLRP3* gene mutations. These mutations lead to constitutive activation of NLRP3 inflammasome, increasing the activity of caspase-1 and the expression of IL-1β and IL-18 [100]. CAPS patients can be effectively treated with therapies targeting IL-1, which confirms its pathogenic role [101]. 

CAPS include familial cold autoinflammatory syndrome (FCAS), Muckle–Wells syndrome (MWS), and neonatal-onset multisystem inflammatory disease [102,103]. The clinical manifestations of these diseases vary widely, ranging from fever to systemic inflammatory conditions and dermal eruptions, in which mast cells play a significant role [103]. A study on mouse myeloid cells conducted by Brydges et al. showed that mutations of *Nlrp3^A350V^* (A352V) and Nlrp3^L351P^ (L353P) were associated with the pathogenesis of FCAS and MWS, respectively [104]. Research on CAPS undertaken by several groups focused primarily on the mechanisms of inflammasome activation in monocytes, macrophages, and dendritic cells. However, none of the studies so far identified the cell group that plays the key role in the pathogenesis of CAPS [11,105,106]. Notwithstanding, the research by Hoffman and Ley, which analyzed the role of neutrophils in CAPS, demonstrated that neutrophilia and the infiltration of PMNs in tissues are characteristic of CAPS, following which studies on the importance of neutrophils in this disease group were expanded [107,108]. Stackowich et al. studied at the mRNA level the genes responsible for the hyperactivity of NLRP3 and IL-1β inflammasome in different populations of mouse cells and identified neutrophils, dendritic cells, monocytes, and basophils as potential sources of hyperactive NLRP3, excluding mast cells. Interestingly, the authors pointed out neutrophils as the main source of IL-1β among the 21 types of immune cells analyzed. Considerable expression of IL-1β of neutrophil origin was also observed in the dermal infiltrates of patients with CAPS and in a mouse CAPS model (mutation of *Nlrp3 ^A350V^*), which confirmed the contribution of PMNs in the clinical manifestations of these syndromes [109]. The results of the above study suggest that neutrophils could be a potential therapeutic target in the treatment of the clinical manifestations of CAPS. CXCR2 receptor antagonists for chemokines on neutrophils are a potential grip point for reducing the neutrophil count [110]. Clinical studies on CXCR2 blocking antibodies were conducted, among others, for asthma [111]. Further research on the treatment of CAPS symptoms is undoubtedly needed to explain the pathogenic mechanism involving neutrophils, although PMNs are certainly promising candidates for targeted therapy of these diseases.

## 5. Summary and Future Prospects

Neutrophils exhibit their own cellular identity, which has an effect on their various effector functions. The inflammasome activation-related signaling of these cells, which differs from that of monocytes/macrophages, is the best proof of their distinct nature. The mechanisms underlying the activation of protein complexes and the expression of IL-1β neutrophils remain unclear. It is essential to understand the activation mechanism of inflammasomes and their role in various clinical entities, as they could be significant therapeutic targets with the potential to reduce inflammation in numerous diseases [112].

Despite their significantly higher count compared to monocytes, PMNs are often overlooked when explaining the significance of inflammasome activation in the pathogenesis of different diseases, including gout or neutrophilic dermatoses [113,114]. The contribution of PMNs in the course of these diseases is indisputable, yet studies continue to focus only on inflammasome activation in macrophages. There is a need for a better understanding of the pathogenic mechanisms of inflammatory conditions associated with the activation of inflammasomes in neutrophils. Regulation of the counts of neutrophils, their recruitment, or the use of inhibitors affecting their effector functions could be potential therapeutic approaches. Although medications affecting the function and counts of neutrophils are being sought continuously, they cannot be utilized without a thorough understanding of the contribution of neutrophils to pathogenic mechanisms [110]. Thus, the need for expanding the research on the effector functions of neutrophils and their role in the pathogenesis of inflammatory conditions appears to be justified.

## Data Availability

Not applicable.
